# Quantitative differences in synthetic gut microbial inoculums do not affect the final stabilized *in vitro* community compositions

**DOI:** 10.1128/msystems.01249-22

**Published:** 2023-07-10

**Authors:** Thiyagarajan Gnanasekaran, Arjun Sarathi, Qing Fang, Asieh Azarm, Juliana Assis Geraldo, Eleonora Nigro, Manimozhiyan Arumugam

**Affiliations:** 1 Novo Nordisk Foundation Center for Basic Metabolic Research, Faculty of Health and Medical Sciences, University of Copenhagen, Copenhagen, Denmark; Universita degli Studi di Trento, Povo, Trento, Italy

**Keywords:** synthetic gut microbial community, *in vitro* gut models, SHIME

## Abstract

*In vitro* studies of synthetic gut microbial communities (SGMCs) can provide valuable insights into the ecological structure and function of gut microbiota. However, the importance of the quantitative composition of an SGMC inoculum and its effect on the eventual stable *in vitro* microbial community has not been studied. To address this, we constructed two 114-member SGMCs differing only in their quantitative composition—one reflecting the average human fecal microbiome and another mixed in equal proportions based on cell counts. We inoculated each in an automated anaerobic multi-stage *in vitro* gut fermentor simulating two different colonic conditions, mimicking proximal and distal colons. We replicated this setup with two different nutrient media, periodically sampled the cultures for 27 days, and profiled their microbiome compositions using 16S rRNA gene amplicon sequencing. While nutrient medium explained 36% of the variance in microbiome composition, initial inoculum composition failed to show a statistically significant effect. Under all four conditions, paired fecal and equal SGMC inoculums converged to reach stable community compositions resembling each other. Our results have broad implications for simplifying *in vitro* SGMC investigations.

**IMPORTANCE:**

*In vitro* cultivation of synthetic gut microbial communities (SGMCs) can provide valuable insights into the ecological structure and function of gut microbiota. However, it is currently not known whether the quantitative composition of the initial inoculum can influence the eventual stable *in vitro* community structure. Hence, using two SGMC inoculums consisting of 114 unique species mixed in either equal proportions (Eq inoculum) or resembling proportions in an average human fecal microbiome (Fec inoculum), we show that initial inoculum compositions did not influence the final stable community structure in a multi-stage *in vitro* gut fermentor. Under two different nutrient media and two different colon conditions (proximal and distal), both Fec and Eq communities converged to resemble each other’s community structure. Our results suggest that the time-consuming preparation of SGMC inoculums may not be needed and has broad implications for *in vitro* SGMC studies.

## INTRODUCTION

Human gut microbial communities that exist in nature are very heterogeneous and possess complex interaction patterns giving rise to a myriad of functions (1). They are associated with numerous diseases, where the direction of causality is often difficult to establish (2). We have a very limited understanding of the fundamental ecological and evolutionary aspects of natural microbial communities underpinning ecosystem functions, e.g., the role of the gut microbiome in human health (3). One way to overcome this limitation is to construct synthetic gut microbial communities (SGMCs) that can retain essential features of natural communities, which can then be used to assess ecological, structural, and functional features of communities in a systematic and controlled manner when studied using *in vivo* or *in vitro* model systems (3, 4). Some studies have constructed SGMCs by choosing a few highly prevalent species from the predominant phyla of gut microbiota namely *Firmicutes*, *Bacteroidetes*, *Actinobacteria*, *Proteobacteria*, and *Verrucomicrobia*. This way prominent members of the gut microbial community can be represented, and upon characterization, these SGMCs can yield valuable information that closely matches natural communities (5 - 9). Other studies have built communities that can specifically perform desired functions such as counteracting pathogens (7, 10, 11), producing health-relevant metabolites beneficial to the host (5, 12), and deriving mixture of probiotics (13). Based on the prevalence and relative abundance profiles of strains in the human gut microbiome (14), a recent study constructed a synthetic community of 104 strains, which was further developed into a second community of 119 strains that could resist colonization of pathogenic *Escherichia coli* (15).

When preparing SGMC inoculums, most studies mix the individual strains at approximately equal abundances, usually inferred by their optical density (OD) measurements, as this simplifies the preparation (7, 8, 15). However, an open question is whether strain relative abundances in the inoculum influence the community structure as the experiment progresses and a stable community composition is reached. To answer this question, we constructed two defined SGMCs consisting of the same member species but at different proportions, cultured them in two different growth media using the Simulator of the Human Intestinal Microbial Ecosystem (SHIME) *in vitro* gut model (16 - 20), and characterized these microbial communities over 27 days. We assessed how these initial conditions influenced the communities as they reached stable compositions.

## RESULTS AND DISCUSSION

### Construction of SGMC inoculums and *in vitro* culturing in a multi-stage gut model

**Fig 1:**
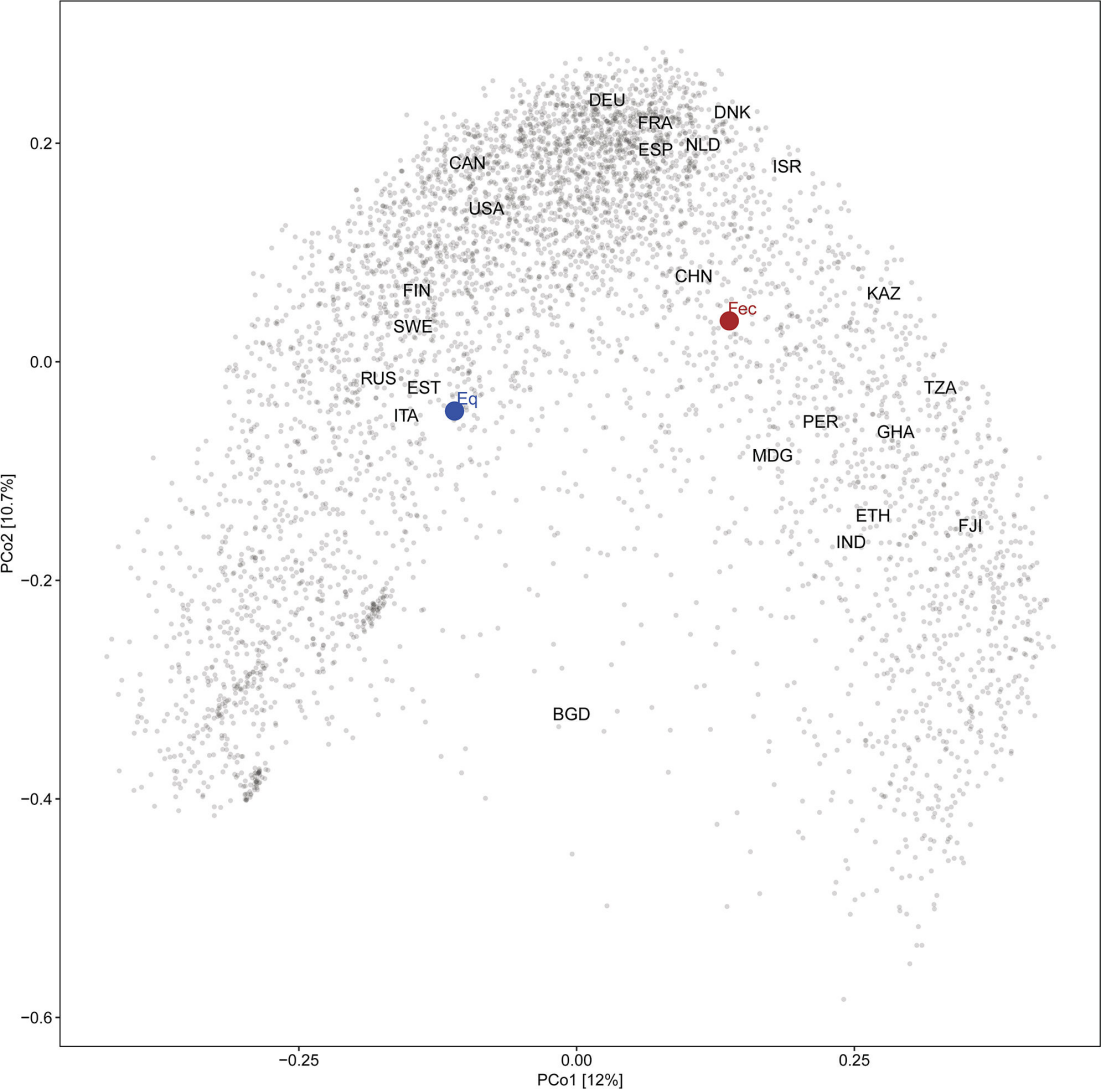
Principal coordinate analysis (PCoA) using Bray-Curtis dissimilarity shows the composition of two theoretical SGMCs used in this study and 5,084 healthy fecal samples from 23 different countries. Abbreviated names of countries denote the mean country-specific microbiome compositions (Table S1).

We selected 114 bacterial species by augmenting a previously published list of 59 gut microbial species (21) with 55 additional species of interest. We then estimated the average abundance of each of the 114 selected species using 5,084 healthy fecal microbiome samples (spanning 23 countries published between 2012 and 2019) to model multiple basal microbiomes from distinct populations (Table S1) (22). To control the bias due to the highly variable number of samples from each country, we first estimated average microbiome compositions for each country and then averaged these for the final estimate (Fig. 1). Using these estimates, we defined the theoretical abundances of the 114 strains and constructed a theoretical SGMC that we named “Fecal (Fec)” (Table S2). Simultaneously, we also derived another SGMC with the same 114 bacterial species but constituting equal abundances that we named “Equal (Eq).”

One drawback was that the Fec SGMC represented a centroid composition of 23 countries and did not precisely reproduce the composition of a specific naturally existing fecal microbial community from an individual, a single country, or even a continent. However, using the centroid allowed us to include most strains from our in-house bacterial collection, thereby increasing the community complexity. Even when inoculating the *in vitro* model system with a real human fecal sample, the *in vitro* culture does not resemble a realistic human fecal microbiome (20). The same could be said about transplanting human feces into germ-free mice. Nevertheless, they are both valuable models to understand the ecology of the human gut microbiome, to perform specific interventions/perturbations, and to establish causality. Thus, although Fec and Eq SGMCs are not real representative fecal microbial communities, both are complex ecosystems (with 114 species) and their quantitative differences (Fig. 1) are adequate to investigate whether species relative abundances in the inoculum influence the final stabilized community structure.

To construct *in vitro* Fec and Eq SGMC inoculums, we cultivated axenic cultures of the 114 species in their respective media (Materials and Methods) and mixed them according to the theoretical Fec and Eq abundances using their cell count information (Table S3). Most *in vitro* experiments involving predefined bacterial communities have used OD measurements for deriving the starter inoculums (8, 9, 15). Although OD values are easy to measure, they may not relate linearly to the cell count except for a limited range that is fairly different from one species to the other (23). To verify this, we performed cell counting as well as OD measurements at 600 nm (OD_600_) for all 114 axenic cultures and created 114 pairs of OD_600_ and cell counts. We found that these pairings were highly individual and not even transferable between bacterial species within the same genera. For instance, *Bacteroides eggerthii* and *Bacteroides intestinalis* with OD_600_ of 1.89 and 1.91 had cell counts of 4.6 × 10^7^ and 2.3 × 10^7^, respectively; *Phocaeicola massiliensis* and *Phocaeicola plebius* with OD_600_ of 1.75 and 1.68 had cell counts of 9.0 × 10^7^ and 1.4 × 10^7^, respectively (Table S3). Therefore, we mixed the individual monocultures purely based on cell counts to reliably derive the SGMC inoculums in defined proportions and anticipated that our cell-count-adjusted Eq and Fec *in vitro* inoculums better represent the target inoculums.

To culture the SGMCs, we used the luminal QuadSHIME (24, 25) setup with four parallel units, wherein each unit comprised a combined stomach and small intestine compartment, a proximal colon (PC) compartment, and a distal colon (DC) compartment (Fig. S1). This allowed us to culture the two SGMCs each in two different nutrient media. Along with the standard feed (SF) that is commonly used in SHIME experiments (20, 26, 27), we formulated a novel nutrient medium that contained brain heart infusion (BHI) medium, SF and cooked meat medium (CMM) in the proportion 45:45:10 (termed BSC hereafter; Materials and Methods). We introduced the two SGMC inoculums into the *in vitro* fermentor, maintained the culture for 27 days, and sampled the colon compartments every second day starting from day 1 (D1).

### Characterizing microbiome compositions via amplicon sequencing needs thorough quality control

We performed 16S ribosomal RNA gene V4 region amplicon sequencing of 112 *in vitro* samples and the two inoculums. As *negative controls*, we sequenced (i) two tubes of sterile media (BSC and SF) and an empty tube that were subjected to the DNA extraction steps along with the inoculums and samples, and (ii) elution buffer that was used in the final step of DNA extraction. We also sequenced two commercial microbial community DNA standards from ZymoBIOMICS as *sequencing controls* (Materials and Methods). By analyzing the amplicon sequence data from these 120 samples using DADA2 software (28), we obtained a total of 449 microbial amplicon sequence variants (ASVs).

Before investigating the dynamics of our SGMC members over time, we verified whether the 114 species could be individually resolved using 16S V4 amplicon sequencing. Using the whole genome sequences of these 114 species, we found that some congeneric species (from *Enterococcus*, *Staphylococcus*, *Lactobacillus*, *Blautia*, and *Bifidobacterium*) had identical sequences in the V4 hypervariable regions in their 16S rRNA genes (Table S4). Hence, the species-level distinction of these bacteria was not possible using our sequencing data. Thus, any computational workflow (including ours) could only resolve 105 taxa (98 resolved at the species level and 7 at the genus level) from the 114 species added to the synthetic communities.

As a first step of quality control, we compared the theoretical and observed abundances of species from both sequencing controls (Fig. S2). In the first microbial community DNA standard consisting of eight bacterial species with 12% DNA from each, their observed relative abundances closely matched the theoretical relative abundances after adjusting for 16S rRNA gene copy numbers (coefficient of determination, *R* ^2^ = 0.87). We also observed five contaminant ASVs at very low abundance: ASVs belonging to the genera *Tepidimonas*, *Megamonas*, *Brevibacillus*, *Micrococcus*, and one ASV belonging to the Family *Paenibacillaceae* (Fig. S2A). In the second standard with the same species varying from 89.1% to 0.000089% relative abundance as a log distribution, goodness of fit after log transformation of relative abundances was low (coefficient of determination, *R* ^2^ = 0.14). This was predominantly due to four contaminant ASVs belonging to the genera *Megamonas*, *Bacteroides*, *Pyramidobacter*, and *Carnobacterium* (Fig. S2B). When we re-estimated the *R* ^2^ discarding these contaminant ASVs, thereby using only the eight species in the standard, the coefficient of determination increased to 0.86. These results suggested that our sequencing workflow closely maintained the relative abundances of species in the microbiome samples. They also highlighted the well-known issue of contaminants in 16S rRNA profiling (29, 30). Indeed, our workflow identified 444 ASVs (after removing five ASVs that were unique to the sequencing standards), which was more than four times the resolvable taxa. We anticipated that a vast majority of these ASVs were likely artifacts or contaminants introduced during *in vitro* experiment, DNA extraction, or sequencing (29, 31).

To obtain a robust set of ASVs from the initial set of 444 ASVs, we sorted them based on their average relative abundance across 112 *in vitro* culture samples and the 2 inoculums and aimed to identify a cutoff below which we could remove ASVs. After manually marking ASVs that can be likely linked to one of the 105 resolvable taxa as true positives, and the remaining ASVs as false positives, we identified that an optimal cutoff at 128 ASVs (covering 75 out of the 105 resolvable taxa) captured most of the inoculated species with minimum contaminant species as evaluated by F1-score (Fig. S3A). In parallel, we also used the decontam package (32), which yielded 131 ASVs after removing contaminants, covering only 63 out of the 105 resolvable taxa. Additionally, while our 128 ASVs explained nearly 100% of the relative abundances in all *in vitro* samples and inoculums (minimum 99.86%), the 131 ASVs from decontam did not fully represent several samples, especially the inoculums and day 1 samples (Fig. S3B). This suggested that decontam mislabeled some ASVs as contaminants when they did not survive in the *in vitro* fermentor. Therefore, we decided to use 128 ASVs from our own procedure for further analysis and merged ASVs from the same species, which resulted in 111 taxa, with 72 taxa resolved at the species level and 33 resolved only at the genus level. Overall, these taxa represented 52 unique genera, 34 unique families, and 8 unique phyla.

Of the 105 resolvable taxa, we could detect 71 in the inoculums at our sequencing depth. While we detected 50 taxa in both inoculums, 4 taxa were unique to the Fec inoculum and 17 taxa to the Eq inoculum, showing that we detected a higher number of taxa in the Eq inoculum that had an even composition. Among the 71 taxa, 21 taxa went below the detection threshold as the experiment progressed, leaving 46 and 47 taxa detected in Fec and Eq *in vitro* cultures. On the other hand, seven new taxa that were not detected in the inoculums emerged during the experiment.

### Bacterial abundance differences in inoculum do not affect the stabilized community composition

Though we detected 54 and 67 taxa in the Fec and Eq inoculums, respectively, the *in vitro* cultures exhibited reduced ASV richness on D1: the compartments fed with SF medium (SF-fed) exhibited richness between 21 and 25, and those fed with BSC medium (BSC-fed) exhibited richness between 30 and 37. Most compartments maintained this reduced richness in a stable manner except SF-fed DC, where the richness increased before stabilizing. Throughout the experiment, the richness in SF-fed PC stayed lower than the SF-fed DC, while the richness of BSC-fed PC and BSC-fed DC remained comparable (Fig. S4A). We hypothesize that BSC as a nutrient-rich medium might support more bacteria even at a lower pH. Irrespective of the colon compartments, richness reached near-stable values from D3 onwards suggesting that the inoculum differences did not affect how the community richness stabilized over time. However, in the case of Shannon diversity, all the BSC-fed compartments exhibited near stable values starting from D3, while the SF-fed compartments exhibited a slightly decreasing trend till D9 and a gradually increasing trend from D9 to D29 (Fig. S4A). This phenomenon of early stabilization (from D3) of ASV richness was surprising, as previous studies have shown that it usually takes at least a week for richness to reach near-stable values in typical experiments with SHIME that used inoculum prepared directly from feces (20, 24).

**Fig 2:**
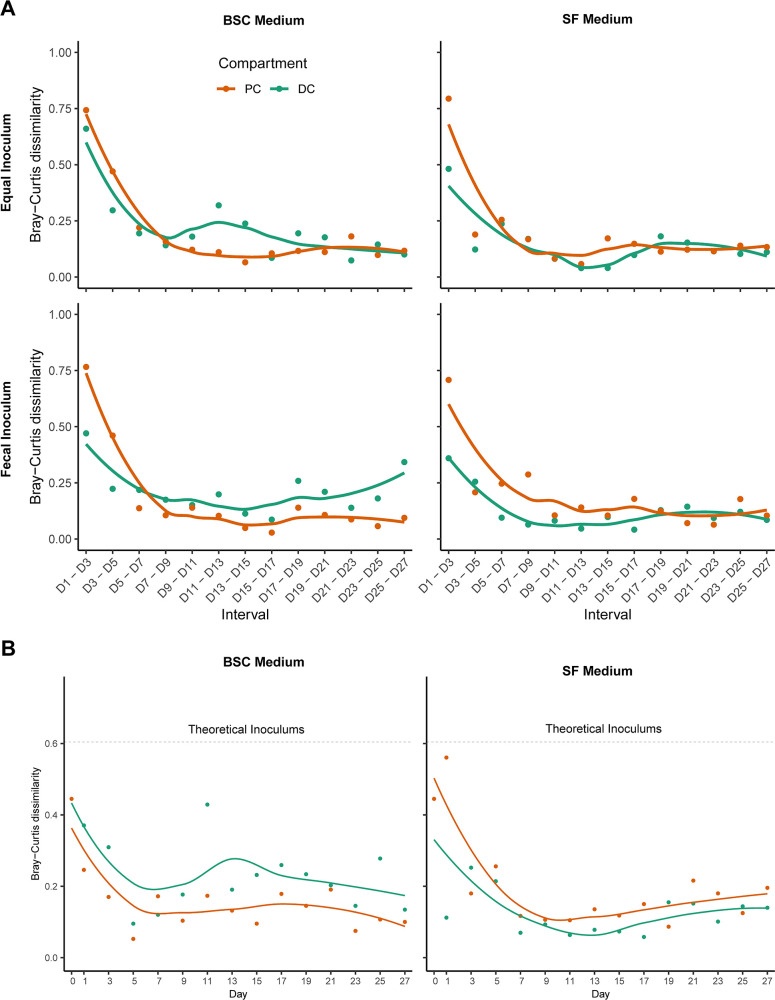
Stabilization and convergence of SGMCs. (**A**) Microbiome composition differences between consecutive time points (2-day intervals) show stabilization by D11 in all eight compartments (PC/DC compartments with Fec/Eq inoculums fed with BSC/SF media). (**B**) Convergence of Fec and Eq community compositions under matched medium and colon compartment combinations. Theoretical composition difference between Fec and Eq inoculums is shown for reference. PC compartment is denoted by orange points and lines, and DC compartment is denoted by green points and lines. Bray-Curtis dissimilarity was used as beta diversity measure.

We then investigated the longitudinal changes in community composition using Bray-Curtis dissimilarity as the beta diversity measure. In the combined principal coordinate analysis, the first principal coordinate separated samples by the medium (Fig. S4B). Within each compartment, variation in microbiome compositions between consecutive samples (2-day intervals) reached near-stable values around D11 for both BSC-fed and SF-fed compartments after exhibiting a sharp decreasing trend till D9 (Fig. 2A). At the same time, for each medium-compartment combination, Fec and Eq community compositions converged toward each other—estimated beta diversity between Fec and Eq communities from matching medium-compartment combinations dropped over the first week and stabilized below 0.2 (Fig. 2B), which was comparable to the stable 2-day variation within each compartment (Fig. 2A). To provide a reference scale, we also estimated Bray-Curtis dissimilarity between theoretical Eq and Fec inoculums. Estimated beta-diversity between Fec and Eq communities from the colon compartments converged to much lower values compared to the difference between theoretical inoculums and actual inoculums (marked as Day 0 in Fig. 2B).

**Fig 3:**
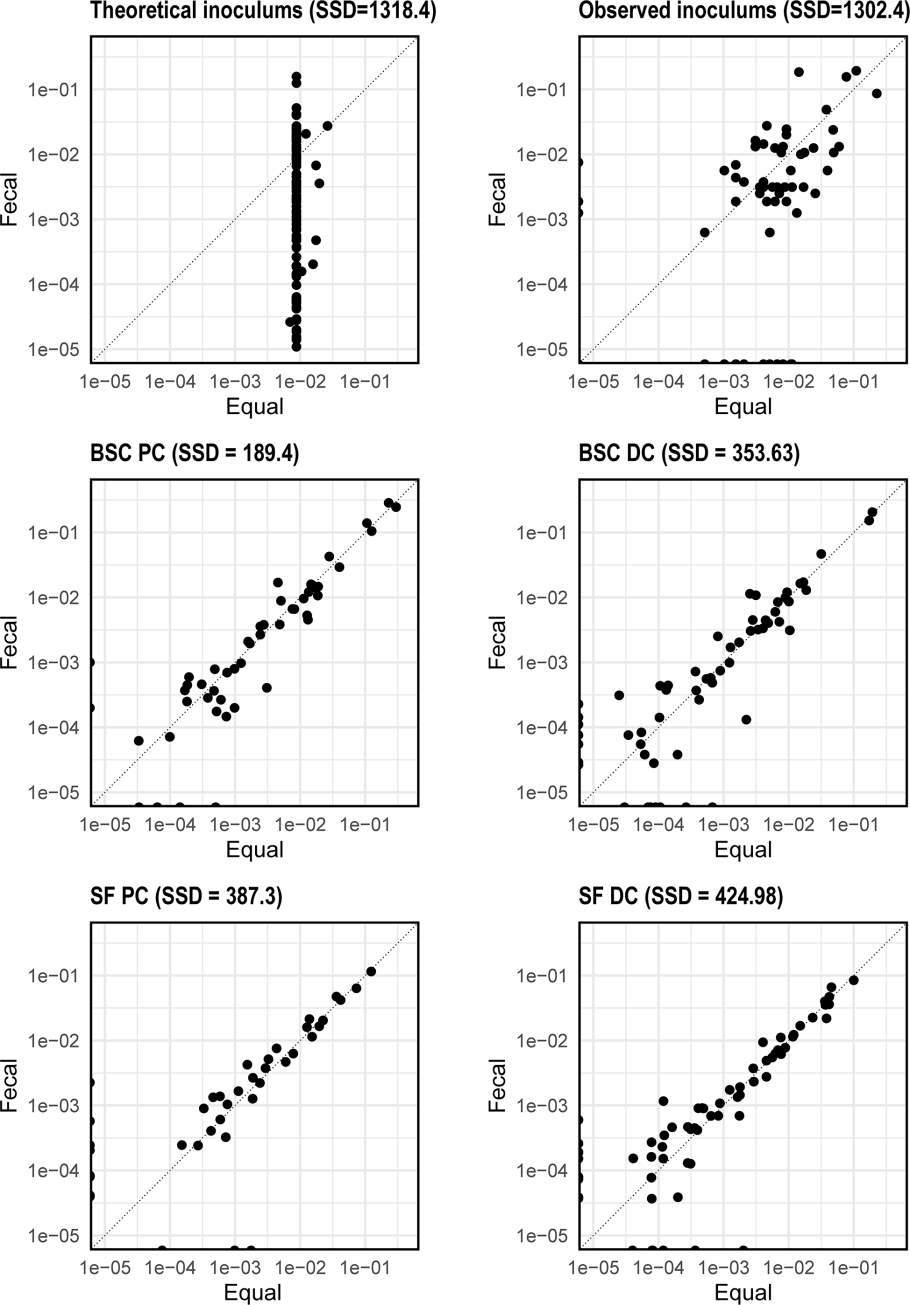
Comparing individual relative abundances of species in the fecal and equal SGMCs. Top row compares theoretical and observed inoculums. Second and third rows compare SGMCs grown in BSC and SF media, respectively. Sum of squared differences for each plot are also listed.

To further investigate this convergence, we measured the deviation between Fec and Eq communities using the sum of squared difference (SSD) in ASV relative abundances. We first derived the average stable community composition from the post-stabilization samples (D13–D29) in each compartment. We then estimated SSD between the stable Fec and Eq communities for matched compartments (Fig. 3). Measured SSDs between stable Fec and Eq communities from the colon compartments were much lower (below 425 units) compared to the difference between inoculums (theoretical inoculums: 1,318 units; observed inoculums: 1,302 units), irrespective of the medium used (Fig. 3). They were also in a similar range as the community differences between samples obtained from two consecutive time points (Fig. S5A). Looking at the longitudinal trends in SSDs, Fec vs Eq communities quickly converged within the first 5 days and maintained low SSD throughout the rest of the experiment (Fig. S5A and Fig. S5B), mirroring the results using Bray-Curtis dissimilarity measure.

### Growth media influence microbiome composition in a compartment-specific manner

**Fig 4:**
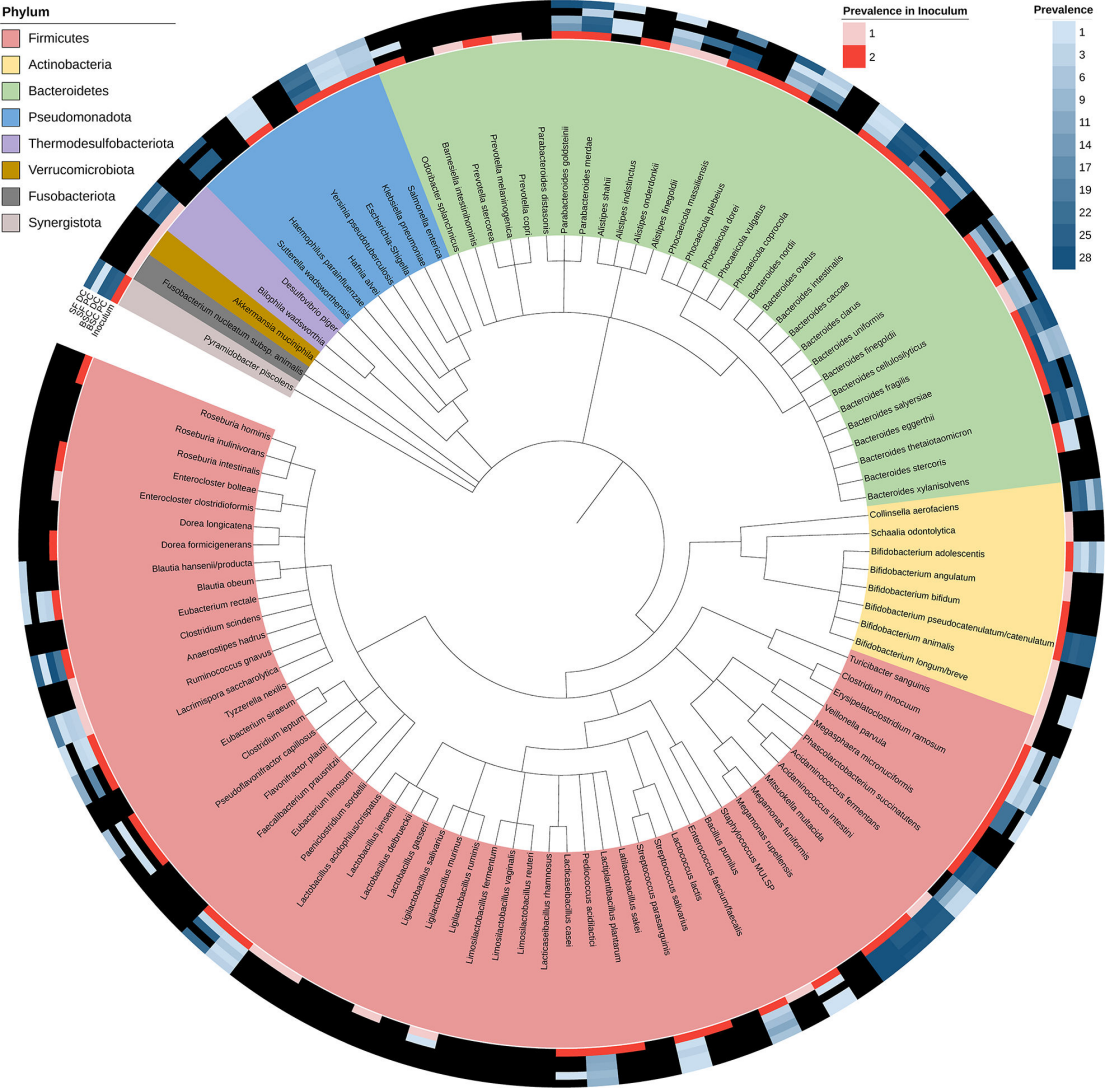
Detection patterns of the original inoculum taxa under different *in vitro* conditions. Phylogenetic tree of the 105 resolvable taxa that were part of the original inoculum is shown. Taxa are colored by their phylum affiliation; outer rings show the frequency of their detection in the inoculums, SF-fed and BSC-fed PC/DC compartments, across all time points. Black cells in the outer rings represent lack of detection. Species that are not distinguishable by their 16S rRNA V4 variable region sequence have been merged (see Table S4).

Out of the 105 resolvable taxa, *Acidaminococcus fermentans*, *Alistipes shahii*, *Bacteroides thetaiotaomicron*, *Blautia hansenii/producta*, *Collinsella aerofaciens*, *Phocaeicola vulgatus*, and *Sutterella wadsworthensis* were below our detection threshold in both inoculums but were observed in the *in vitro* environment (Fig. 4). These species likely flourished due to favorable nutrient and environmental conditions in the *in vitro* fermentor similar to the bloomer species that we reported recently, which were below our detection threshold in the fecal sample but flourished in the *in vitro* environment (20).

Although the quantitative differences in relative abundances of starting inoculums did not alter the stabilized community composition, a clear difference in the community composition was observed based on the growth medium used (Fig. S4B). Permutational analysis of variance (PERMANOVA) analysis using Bray-Curtis dissimilarity (Table S5) showed that medium (SF or BSC) explained the largest variance in ASV composition (*R* ^2^ = 38.6%, *P* = 0.001) followed by compartment (PC or DC) explaining 6.7% variance (*P* = 0.001). Inoculum type (Fec or Eq) did not significantly explain the variance in ASV composition (*R* ^2^ = 0.4%, *P* = 0.56).

Among the 57 ASVs detected in the *in vitro* culture samples, 47 were detected in both BSC- and SF-fed compartments. When we looked for media-specific ASV patterns, *Alistipes finegoldii*, *Bacteroides finegoldii*, *Bacteroides stercoris*, and *Pseudoflavonifractor capillosus* were detected in at least two samples from BSC-fed compartments while the same species were not detected in SF-fed compartments. On the other hand, *Bifidobacterium longum/breve* and *Blautia hansenii/producta* were detected in at least two samples from SF-fed compartments but not in BSC-fed compartments.

Next, we looked for ASV patterns in PC vs DC compartment comparisons. Among the 57 ASVs detected in the *in vitro* culture samples, 54 were detected in DC compartments and 49 in PC compartments. This difference was more pronounced in the SF-fed compartments: SF-DC harbored 18 more ASVs than SF-PC (49 vs 33), while BSC-DC harbored only 4 more ASVs than BSC-PC (51 vs 47). On the other hand, we did not identify any PC-specific ASVs. *Clostridium leptum* and *Tyzzerella nexilis* were detected in both DC compartments but in none of the PC compartments indicating that irrespective of the nutrient differences between SF and BSC, close-to-neutral pH is vital for the successful growth of these bacteria.

Furthermore, *Alistipes shahii*, *Clostridium innocuum*, *Eubacterium limosum*, *Lacrimispora saccharolytica*, *Veillonella parvula*, *Bacteroides uniformis*, *Bacteroides eggerthii*, *Phocaeicola massiliensis*, *Alistipes onderdonkii*, *Parabacteroides goldsteinii*, *Sutterella wadsworthensis*, *Bacteroides cellulosilyticus*, *Bacteroides intestinalis*, and *Blautia obeum* were not detected in the SF-fed PC compartments but successfully colonized in all the other compartments (Fig. 4). The selective enrichment of these bacteria in the BSC-fed PC but not in the SF-fed PC demonstrates the significance of the growth medium differences despite the similarity in pH. We anticipate that the animal-derived nutrients present in BSC might have played a vital role in the successful growth of these bacteria at low pH.

SF-fed compartments also selectively enriched *Bacteroides cellulosilyticus*, which was detected in higher abundances in SF-fed DC compared to other compartments. This is not surprising as SF is comparatively rich in plant-derived glycans, and this bacterium is known to be cellulolytic (33).

Among the 10 most abundant species in the theoretical Fec inoculum (Table S2), *Prevotella copri*, *Faecalibacterium prausnitzii, Eubacterium rectale*, and *Akkermansia muciniphila*, which accounted for 14%, 11%, 9%, and 2% in relative abundance, respectively, were not detected in the *in vitro* setup. This is very surprising, as previous studies (20, 34) have shown that these bacteria could successfully colonize SHIME when SF was used as the nutrient medium. The exact reason why these bacteria could not colonize in the current study is not clear. The source of the inoculum could influence the colonization preferences in the *in vitro* setup as we have used artificially derived SGMCs using isolates from diverse sources, while the other two studies used inoculums directly derived from human feces. Factors such as strain differences and other outcompeting bacteria could have also played a role.

### Conclusions

Large SGMCs with complex composition dynamics are promising avenues for therapeutic modulation of human gut microbiota (35). In this work, we constructed two large SGMCs (114 bacterial species) to decipher the effect of inoculum differences on the stabilized community composition in an *in vitro* gut model. Our results suggest that despite the quantitative differences in abundances of SGMC inoculums, the communities converged toward similar compositions, suggesting that it may not be crucial to precisely quantify and mix bacterial species in defined proportions to make SGMC inoculums. Thus, our results support the simplified construction of SGMCs, especially for the studies involving *in vitro* gut models. Further investigations using additional media and inoculum compositions could investigate if our results could be generalized.

## MATERIALS AND METHODS

### Procurement and cultivation of bacterial strains

All the bacterial strains (Table S2) were purchased either as lyophilized powders or as actively growing anaerobic cultures from the German collection of microorganisms and cell cultures GmbH (DSMZ, Braunschweig, Germany). The rehydration of lyophilized strains and the inoculation of the actively growing cultures were performed under anoxic conditions inside the anaerobic chamber (Coy Laboratory Products, Ann Arbor, MI, USA) containing 85% N_2_, 10% CO_2_, and 5% H_2_. For the lyophilized strains supplied in glass ampoules, 0.5 mL of anoxic medium recommended by DSMZ for each strain was added to the inner vials containing the lyophilized powders and incubated for 30 min. The incubated suspension was then added to culture tubes containing 5–10 mL of recommended cultivation media and incubated at 37°C in the anaerobic chamber for 24–72 h. For the actively growing cultures purchased from DSMZ, 1 mL of the cultures was transferred to the culture tubes containing 5–10 mL of recommended cultivation media and incubated at 37°C in the anaerobic chamber for 24–72 h. Upon successful growth of these strains, the cultures were cryopreserved (20% glycerol final concentration) at −80°C until further use.

### Total cell counting and OD measurement

The cryostocks of these bacterial strains were inoculated in one of these media: BHI, Gifu anaerobic medium (Nissui Pharmaceutical, Tokyo, Japan), Man, Rogosa & Sharpe broth (Oxoid Ltd, Basingstoke, United Kingdom), or Standard feed (ProDigest BVBA, Ghent, Belgium) that has been extensively used in the *in vitro* gut fermentor-simulator of the Human Intestinal Microbial Ecosystem (SHIME; Prodigest BVBA, Ghent, Belgium) (20, 26, 27). Total cell counting was performed on the second passage using an impedance flow cytometer BactoBox (SBT Instruments, Herlev, Denmark). For each of the bacterial cultures, serial dilution series were conducted by using 6 mL vials containing a volume of 3 mL 1/20 phosphate-buffered saline buffer until the final concentration was within the detection range (10^4^–10^6^ bacteria/mL). Simultaneously, all the bacterial cultures were diluted 10 times and subjected to OD_600_ measurement using a microplate Epoch 2 spectrophotometer (BioTek, Winooski, Vermont, USA).

### SHIME experimental setup

The *in vitro* gut fermentor SHIME (19) with the luminal Quad-SHIME configuration was employed for the *in vitro* experiment. Each unit of SHIME comprised a combined stomach and small intestine (ST + SI) compartment, a PC compartment, and a DC compartment (Fig. S1). The combined ST + SI compartments operated on a fill-and-draw principle and simulated the digestion, while the colon compartments were continuously stirred fed-batch reactors in which the SGMCs were inoculated. Both the colon compartments were maintained at constant pH and volume—PC: pH 5.6–5.9, v = 500 mL; DC: pH 6.6–6.9, v = 800 mL. During each feeding cycle, 140 mL of feed was added to the ST + SI compartment and incubated for 60 min, followed by the addition of 60 mL of pancreatic juice (PJ) and incubation for an additional 75 min. Then the feed and PJ mix from ST + SI were transferred to DC via PC. These liquid transfer cycles were programmed to take place every 8 h (i.e., three cycles/day). During the entire experiment, all the compartments were maintained at 37°C and flushed with 100% N_2_ for 10 min every day.

### Nutrient medium compositions of the *in vitro* gut fermentor

For the first nutrient medium, we chose SF consisting of arabinogalactan (1.2 g/L), pectin (2 g/L), xylan (0.5 g/L), glucose (0.4 g/L), yeast extract (3 g/L), special peptone (1 g/L), mucin (3 g/L), L-cysteine-HCl (0.5 g/L), and starch (4 g/L). We formulated a second nutrient medium rich in digest/extracts from animal source: SF supplemented with BHI (Oxoid Ltd, Basingstoke, United Kingdom) and CMM ( Oxoid Ltd, Basingstoke, United Kingdom) in the proportion of BHI:SF:CMM = 45:45:10, which we named as BSC. We specifically chose BHI and CMM over other commercially available media, as the BHI/CMM mix as a nutrient media has been shown to support a wider variety of anaerobic bacteria (36, 37). Prior to use, both feeds were adjusted to pH 2. PJ contained NaHCO_3_ (12.5 g/L) (Sigma-Aldrich, St. Louis, Missouri, USA), oxgall (6 g/L) (ProDigest BVBA, Ghent, Belgium), and pancreatin (0.9 g/L) (ProDigest BVBA, Ghent, Belgium). Both the feeds and PJ were maintained at 4°C throughout the experimental period.

### Inoculum preparation, inoculation, and sampling in *in vitro* fermentor

For inoculation of colon compartments of the *in vitro* gut fermentor, we derived SGMCs by mixing 114 axenic bacterial cultures in 2 different proportions (Fec and Eq) based on total cell counts (Table S3). To facilitate mixing, initially, all 114 axenic bacterial cultures were diluted with BHI to obtain an equal cell concentration of 10^5^ cells/mL. We chose 10^5^ cells/mL as it was the lowest cell concentration recorded among the 114 bacterial cultures (*Eubacterium siraeum*; Table S3). We needed at least 130 mL each of Fec and Eq inocula to seed four colon compartments. First, the Eq inoculum was constructed by transferring and mixing 1,300 µL of liquid culture from each of the 114 diluted bacterial cultures in 50 mL propylene tubes (Corning, Corning, NY, USA). This resulting Eq inoculum of 148.2 mL contained a total cell concentration of 1.48 × 10^7^ cells/mL (Table S3). Next, the Fec inoculum with the same total cell concentration as the Eq inoculum was constructed by mixing individual cultures according to the volumes estimated based on the calculated Fec proportions (Table S2 and S3). For all the cultures that required less than 2 µL of volume in the Fec inoculum, a fixed volume of 2 µL was added to avoid pipetting error, reaching a total volume of 147.9 mL. The resulting Fec and Eq mixes were centrifuged at 6,000 rpm for 5 min to remove the supernatant, and the resulting pellet was mixed either with 5 mL of SF or BSC and used for inoculation. Upon inoculation, the SHIME setup was run for 27 days, and the sampling (1 mL) from the colon compartments was performed every second day starting from D1. The collected samples were then centrifuged at 6,000 rpm for 5 min, and both the pellets and supernatants were stored at −80°C for further analysis.

### DNA extraction, procurement of DNA standards, and 16S rRNA gene amplicon sequencing

NucleoSpin Soil DNA kit (Macherey-Nagel, Duren, Germany) was used to extract genomic DNA from the bacterial pellets and negative controls. To lyse the bacterial cells in the pellet, the pellets were resuspended with optional enhancer SX solution and SL1 buffer and lysed using Tissuelyser II (Qiagen, Hilden, Germany) at a speed of 30 oscillations/s for 5 min. The quality of extracted DNA was assessed using NanoDrop and Qubit Fluorometer (Thermo Fisher Scientific, Cleveland, Ohio, USA). The ZymoBIOMICS Microbial Community Standard (D6300) and ZymoBIOMICS Microbial Community Standard II-Log Distribution (D6310) (Zymo Research, Tustin, CA, USA) were used as *sequencing controls*. The ZymoBIOMICS Microbial Community Standard consisted of eight bacteria and fungi in abundances *Listeria monocytogenes*—12%, *Pseudomonas aeruginosa*—12%, *Bacillus subtilis*—12%, *Escherichia coli*—12%, *Salmonella enterica* – 12%, *Lactobacillus fermentum* – 12%, *Enterococcus faecalis* – 12%, *Staphylococcus aureus* – 12%, *Saccharomyces cerevisiae*—2%, and *Cryptococcus neoformans*—2%. ZymoBIOMICS Microbial Community Standard II (Log Distribution) consisted of *Listeria monocytogenes*—89.1%, *Pseudomonas aeruginosa*—8.9%, *Bacillus subtilis*—0.89%, *Saccharomyces cerevisiae*—0.89%, *Escherichia coli*—0.089%, *Salmonella enterica*—0.089%, *Lactobacillus fermentum*—0.0089%, *Enterococcus faecalis*—0.00089%, *Cryptococcus neoformans*—0.00089%, and *Staphylococcus aureus*—0.000089%. The V4 variable region of 16S rRNA genes was sequenced using Illumina HiSeq 2500 (by BGI, Shenzhen, China) producing 2 × 250 bp paired-end reads. In total, 120 samples were sequenced yielding 1,393,042 paired-end reads with 11,600 paired-end reads per sample approximately.

### Bioinformatics analysis

All analyses were carried out using R software (v4.2.0, https://www.R-project.org/).

#### Derivation of fecal inoculum composition

To derive the composition of the “fecal” inoculum, we used the R package curatedMetagenomicData (22). A summary of the data sets is included in Table S1, which spans 5,084 samples across 23 countries. After filtering for samples from individuals marked “healthy” across the data sets used, using the country information, a “mean” microbiome species composition for each country was determined. Subsequently a “mean” of the country means was calculated, to avoid a bias toward countries with more samples. This mean-of-means species composition table was then subset to the 114 species in our strain library and renormalized to sum to 100%.

#### Sequence quality control

DADA2 (v1.24.0) (28) was used with default parameters for the initial processing of reads, which comprised paired Illumina HiSeq amplicon sequencing reads representing the V4 region of the 16S rRNA genes. Primers were removed, and reads were filtered and trimmed according to default parameters. The high-quality filtered and trimmed reads were then dereplicated and merged per sample, and after chimeras were identified and removed, 460 unique ASVs were obtained.

#### Taxonomic assignment

Taxonomy was assigned at the species level with the help of the SILVA database (38), using silva_nr99_v138.1_train_set.fa.gz for annotation until the genus level, and another file silva_species_assignment_v138.1.fa.gz for species level resolution of taxonomic assignment. To improve the assignment of ASVs that were potentially missed at this step, a curated database of the sequences of the 114 inoculum species was used to further improve the species-level assignment of ASVs.

#### Identification and filtering of ASVs

Eleven ASVs that were annotated by SILVA as chloroplast and mitochondria were removed, resulting in a total of 449 bacterial ASVs. When analyzing *in vitro* samples, five standard-specific ASVs were removed that reduced the total number of ASVs to 444.

While testing our ASV selection method to remove contaminants, decontam (32) was run with default parameters, using the “prevalence” method, to compare our results with an existing benchmark.

#### Correction of 16S copy number variation

For all the analyses, normalized values with respect to the 16S copy number for each of the species were used. The copy number information was obtained from the rrnDB database (v5.8) (39). For the ASVs that were only identified at the genus level, we used the median 16S copy number for the respective genus, and for ASVs that are not resolved even at the Genus level, the global median copy number of all other ASVs in our data was used for normalization.

#### Alpha and beta diversity

The phyloseq (v1.40.0) (38) package was used for easy handling of the data, and alpha and beta diversity measures were calculated with the help of this and the vegan (v2.6-2) (40) package.

#### Measure of similarity between samples

In this study, two major measures of similarity were used for the similarity between two samples, the Bray-Curtis dissimilarity and the SSDs.

Bray-Curtis dissimilarity was calculated using the *distance* function in the phyloseq package (38).

The SSD was calculated with a custom function in R, implementing


$$SSD=\sum_{i}{\left(\log\left(Eq_{i}\right)-\log\left(Fec_{i}\right)\right)}^{2}$$

where the iterable *i* is each microbe, *Eq_i_* is the relative abundance of microbe *i* in the equal inoculum condition, and *Fec_i_* is the relative abundance of microbe *i* in the fecal inoculum condition.

#### PERMANOVA

*adonis2* function in the R vegan package (40) was used for the PERMANOVA analysis.

#### Plotting data on a taxonomic tree

To plot data on the taxonomic tree (Fig. 4), Interactive Tree of Life online software (41) was used.

#### Graphing and image generation

Most images were generated in R using the packages ggplot2 (v3.3.6) (42), ggrepel (v0.9.1) (43), and ggpubr (v0.4.0) (44). A variety of packages were used for easier data wrangling: dplyr (v1.0.9) (45), microbiome (v1.18.0) (46), Rcpp (v1.0.8.3) (47, 48), reshape2 (v1.4.4) (49), tidyr (v1.2.0) (50), gtools (v3.9.3) (51), and DECIPHER (2.24.0) (52).

Smoothening of the trends in the plots was done using the *geom_smooth* function in ggplot that used the locally estimated scatterplot smoothing algorithm to generate the curves.

devtools (v2.4.4) (53) was also used for easier installation of certain libraries directly from GitHub.

## Data Availability

Sequencing reads have been deposited at NCBI Short Read Archive under BioProject identifier PRJNA944839. Bioinformatic workflow to reproduce the results is available at GitLab.

## SUPPLEMENTAL MATERIAL

Fig. S1 msystems.01249-22-s0001.pdf QuadSHIME experimental setup used in this study.

Fig. S2 msystems.01249-22-s0002.pdf Comparing theoretical and observed relative abundances of ASVs from the two microbial community DNA standards.

Fig. S3 msystems.01249-22-s0003.pdf Removing contaminant ASVs.

Fig. S4 msystems.01249-22-s0004.pdf Alpha and beta diversity measures in the longitudinal samples.

Fig. S5 msystems.01249-22-s0005.pdf Stabilization and convergence of SGMCs.

Table S1 msystems.01249-22-s0006.xlsx List of metagenomic studies.

Table S2 msystems.01249-22-s0007.xlsx Relative abundances in the fecal inoculum.

Table S3 msystems.01249-22-s0008.xlsx Construction of the Fec and Eq inoculums.

Table S4 msystems.01249-22-s0009.xlsx ASV names for unresolvable species.

Table S5 msystems.01249-22-s0010.xlsx PERMANOVA analysis for effect of compartment, medium, and inoculum.

ASM does not own the copyrights to Supplemental Material that may be linked to, or accessed through, an article. The authors have granted ASM a non-exclusive, world-wide license to publish the Supplemental Material files. Please contact the corresponding author directly for reuse.
