# Supplementary material for: Quantitative differences in synthetic gut microbial inoculums do not affect the final stabilized in vitro community compositions
Source: mSystems. 2023 Jul 10;8(4):e01249-22. doi: 10.1128/msystems.01249-22 (PMC10469597; doi:10.1128/msystems.01249-22)
Supplement: Fig. S1 — QuadSHIME experimental setup used in this study. [file msystems.01249-22-s0001.pdf]

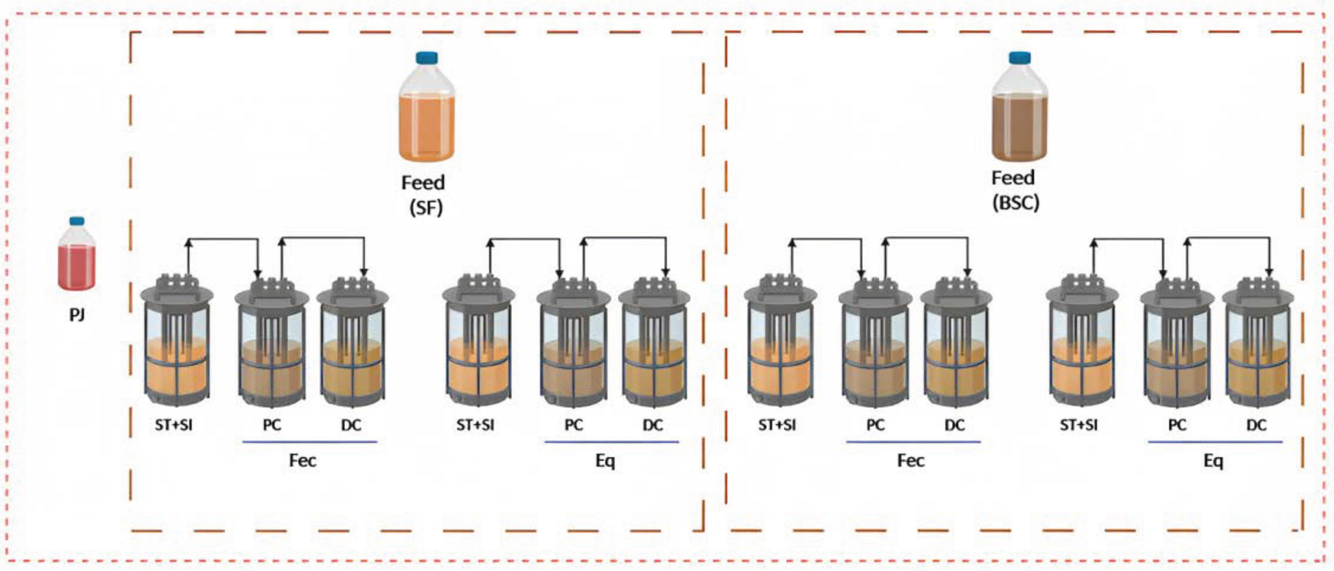

FIG S1 QuadSHIME experimental setup used in this study. SF – Standard feed, BSC – BHI+SF+CMM, PJ – Pancreatic Juice, ST+SI – Combined stomach and small intestine compartment, PC – proximal colon, DC – Distal colon, Fec – inoculum in fecal proportions, and Eq – inoculum in equal proportions. Created with BioRender.com
