# Supplementary material for: Quantitative differences in synthetic gut microbial inoculums do not affect the final stabilized in vitro community compositions
Source: mSystems. 2023 Jul 10;8(4):e01249-22. doi: 10.1128/msystems.01249-22 (PMC10469597; doi:10.1128/msystems.01249-22)
Supplement: Fig. S2 — Comparing theoretical and observed relative abundances of ASVs from the two microbial community DNA standards. [file msystems.01249-22-s0002.pdf]

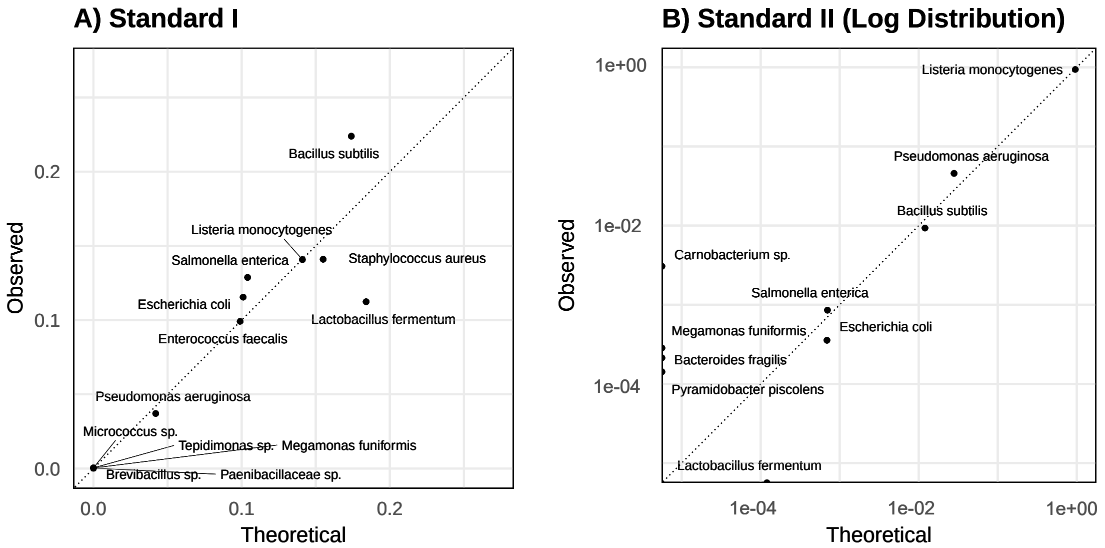

FIG S2 Comparing theoretical and observed relative abundances of ASVs from the two microbial community DNA standards used as sequencing controls (A: equal distribution, B: log distribution).
Theoretical relative abundances are based on the proportion of DNA added to the standards by the supplier. Observed relative abundances for identified ASVs are derived from 16S rRNA gene amplicon sequencing. Both are adjusted for 16S rRNA gene copy number.
