# Supplementary material for: Quantitative differences in synthetic gut microbial inoculums do not affect the final stabilized in vitro community compositions
Source: mSystems. 2023 Jul 10;8(4):e01249-22. doi: 10.1128/msystems.01249-22 (PMC10469597; doi:10.1128/msystems.01249-22)
Supplement: Fig. S3 — Removing contaminant ASVs. [file msystems.01249-22-s0003.pdf]

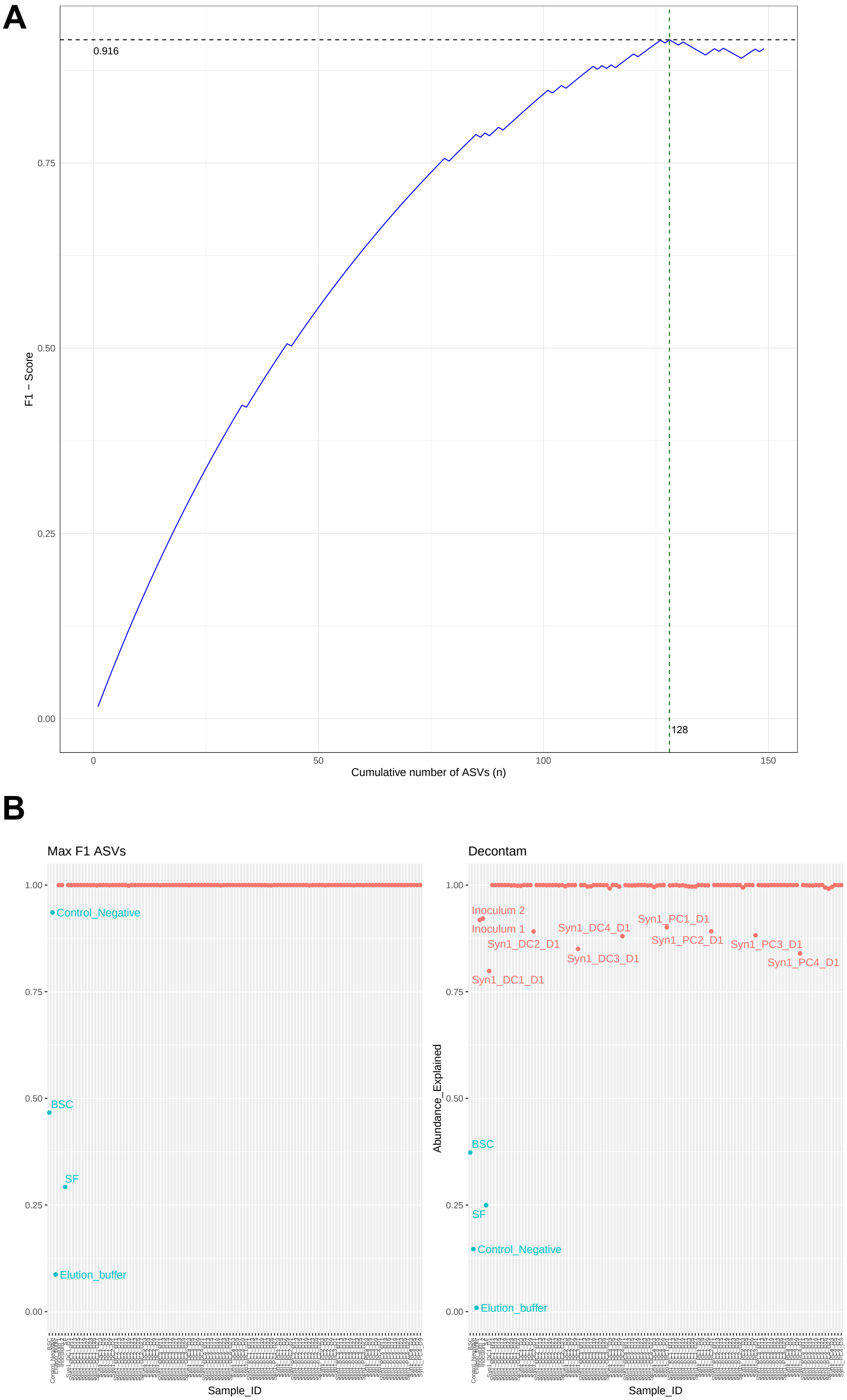

FIG S3 Removing contaminant ASVs. A) The F1 score for ‘n’ ASVs, which is maximized at n = 128, indicating the number of ASVs that maximally explain the composition in positive samples, while excluding those in the negative controls. **B)** Comparison of our method with decontam on choosing ASV sets that contain more information. The set of ASVs that are selected by decontam as relevant did not consider the right abundance values in the early day samples and the inoculums, which is resolved using our method.
