# Supplementary material for: Quantitative differences in synthetic gut microbial inoculums do not affect the final stabilized in vitro community compositions
Source: mSystems. 2023 Jul 10;8(4):e01249-22. doi: 10.1128/msystems.01249-22 (PMC10469597; doi:10.1128/msystems.01249-22)
Supplement: Fig. S4 — Alpha and beta diversity measures in the longitudinal samples. [file msystems.01249-22-s0004.pdf]

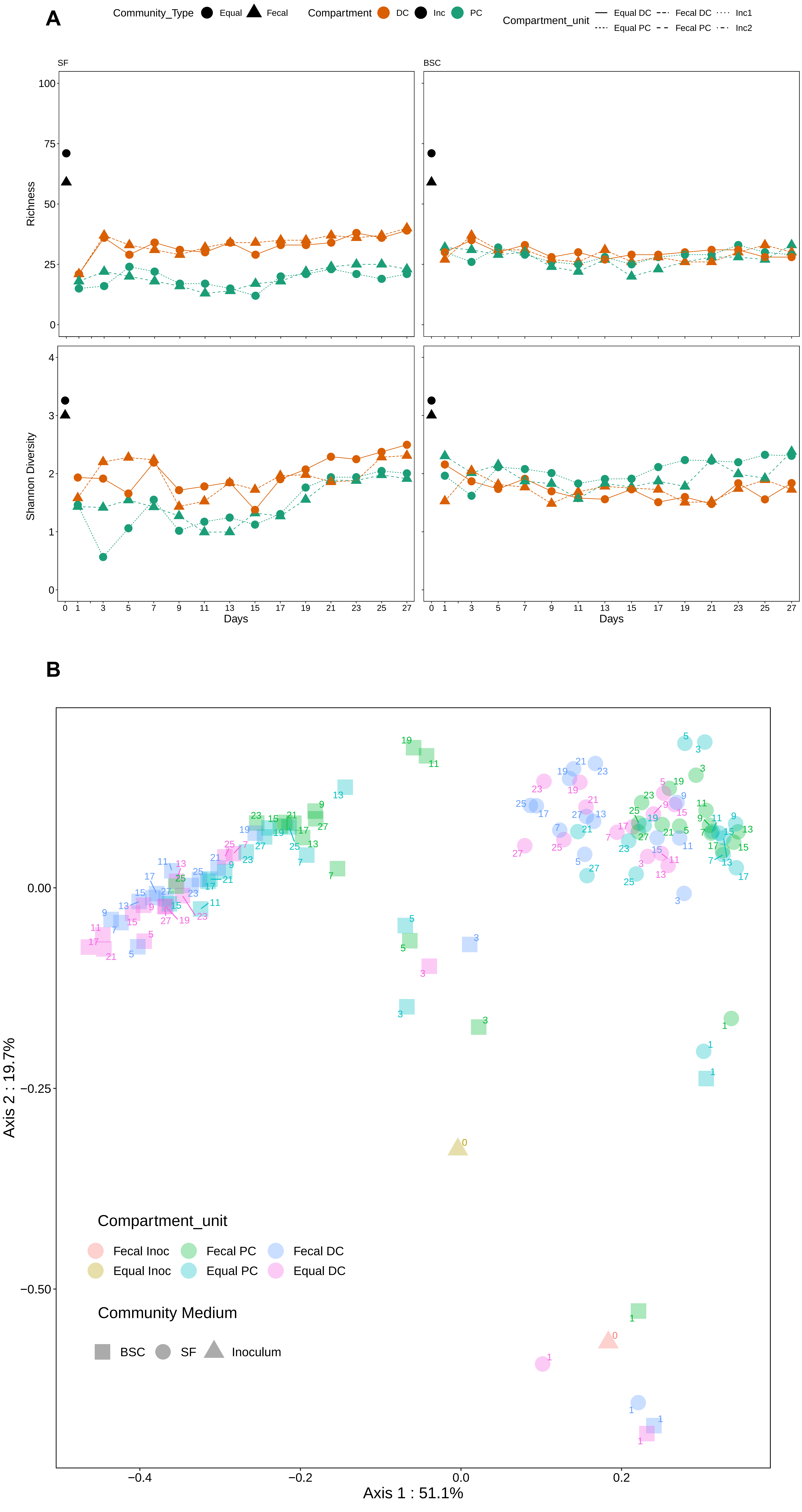

FIG S4 Alpha diversity (**A**) measures (richness and diversity) and beta diversity **(B)** measures observed for the in vitro samples inoculated with Fec and Eq inoculums in SF- and BSC-fed PC and DC compartments.
