# Supplementary material for: Quantitative differences in synthetic gut microbial inoculums do not affect the final stabilized in vitro community compositions
Source: mSystems. 2023 Jul 10;8(4):e01249-22. doi: 10.1128/msystems.01249-22 (PMC10469597; doi:10.1128/msystems.01249-22)
Supplement: Fig. S5 — Stabilization and convergence of SGMCs. [file msystems.01249-22-s0005.pdf]

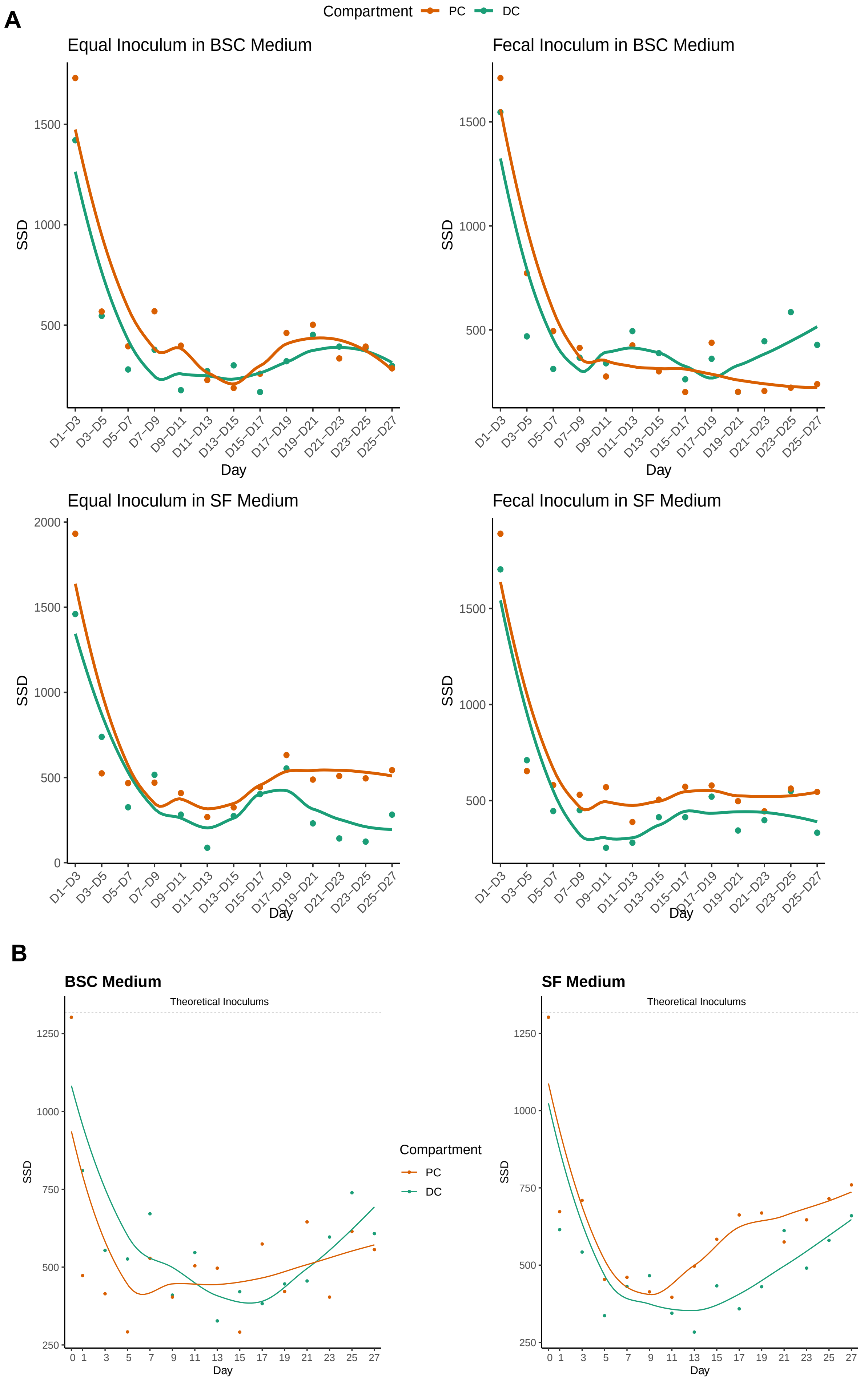

FIG S5 Stabilization and convergence of SGMCs. A) Microbiome composition differences between consecutive time points (2-day intervals) show stabilization by day 11 in all eight compartments (PC/DC compartments with Fec/Eq inoculums fed with BSC/SF media). **B)** Convergence of Fec and Eq community compositions under matched medium and colon compartment combinations. Theoretical composition difference between Fec and Eq inoculums is shown for reference. PC compartment is denoted by orange points and lines and DC compartment is denoted by green points and lines. Sum of squared differences (SSD) was used as beta diversity measure. SF – Standard feed, BSC – Mixed medium consisting of Brain-heart infusion, SF and Cooked meat medium.
